# Initial effectiveness of an ICBT-protocol for GAD in psychiatric care – A feasibility-pilot study

**DOI:** 10.1016/j.invent.2025.100817

**Published:** 2025-03-01

**Authors:** Vilgot Huhn, Erik Andersson, Tove Wahlund, Erik Forsell

**Affiliations:** a Centre for Psychiatry Research, Department of Clinical Neuroscience, Karolinska Institutet, Stockholm, Sweden; b Stockholm Health Care Services, Region Stockholm, Stockholm, Sweden; c Division of Psychology, Department of Clinical Neuroscience, Karolinska Institutet, Stockholm, Sweden

**Keywords:** Generalized anxiety disorder, Routine care, ICBT, CBT, Intolerance of uncertainty

## Abstract

Generalized Anxiety Disorder (GAD) is a common and debilitating anxiety disorder with a chronic course and a low rate of spontaneous remission. Previous internet-delivered treatments for GAD in clinical routine care has been shown to be effective but tend to use a mix of many different treatment components, often based in several theoretical models. Another approach could be to instead develop more focused and theory driven treatments, potentially allowing the protocol to be shorter and less demanding for patients. In this pilot-feasibility-trial we implemented a treatment focusing on one target core construct (intolerance of uncertainty) at the internet psychiatry clinic in Stockholm. The treatment was administered to 22 individuals with GAD. We found a significant reduction in GAD symptoms of similar effect size to previous studies of CBT and ICBT for GAD in routine clinical care. Patients rated overall high levels of treatment satisfaction and treatment credibility. Only two patients dropped out from the treatment. Among the remaining patients a median of 7.5 out of 8 modules were completed. We conclude that the treatment protocol is preliminarily effective, acceptable to patients and clinicians, and feasible to implement in routine psychiatric care.

## Highlights

• A construct-focused treatment protocol for GAD based around the intolerance of uncertainty model was tested within routine psychiatric care.
• Results showed high ratings on treatment satisfaction and treatment credibility.
• Patients had a good level of treatment engagement and low level of drop-out.
• Preliminary estimates of within-group effects show large reductions in self-rated GAD symptoms.
• Future research should verify effectiveness in a larger sample as well as investigate mechanisms of change.

## 1 Introduction

Generalized Anxiety Disorder (GAD) is a psychiatric disorder characterized by excessive worry that is perceived as difficult to control (American Psychiatric Association, 2013). GAD is a common problem with an estimated life-time prevalence of up to 5 % in high-income countries (Ruscio et al., 2017). It is a chronic and debilitating disorder with a low rate of spontaneous remission (Angst and Vollrath, 1991; Ballenger et al., 2001) and has a high degree of comorbidity with other psychiatric disorders (Carter et al., 2001). Previously, the functional impairment associated with GAD was believed to be dependent on these comorbidities, but more recent studies have shown robust associations with impaired occupational and social functioning, as well as a markedly reduced quality of life, even when psychiatric comorbidities are absent (Henning et al., 2007; Hoffman et al., 2008).

Cognitive Behavioural Therapy (CBT) has been established to be effective in treating GAD (Cuijpers et al., 2014), though the treatments appear slightly less effective than CBT for other anxiety disorders (Hunot et al., 2007; Stewart and Chambless, 2009). In recent years, CBT has been successfully tested in an online format (internet-delivered CBT; ICBT) for individuals with GAD in both randomized trials (Richards et al., 2015) as well as in routine clinical care (Hadjistavropoulos et al., 2014; Hobbs et al., 2017; Mewton et al., 2012; Ritola et al., 2022).

Most of these programs have used a broad multi-component approach of interventions which targets several different symptom clusters and comorbid disorders. For instance, Mewton et al. (2012) developed and tested a six-lesson automated ICBT course which included a range of interventions such as behavioral activation, assertiveness skills training and problem solving. Similarly, Ritola et al. (2022) conducted a study on therapist supported ICBT in Finland and adapted a range of different theoretical concepts from metacognitive therapy, the intolerance of uncertainty model of GAD as well as components from the applied relaxation method.

To develop treatments with a few, symptom-specific, therapeutically active components, that are theory- and data-driven has been suggested as a complementary route to the multi-component packages (Andersson et al., 2018; Holmes et al., 2018). It has been argued that the most successful developments in CBT have been in treatments that are based around a theoretical understanding of the core symptoms and the mechanisms that maintain them (Holmes et al., 2018). Focused and theory-driven intervention strategies may thus, in the long term, enable closer investigation of the therapeutically active mechanisms.

This may pose a challenge when it comes to GAD specifically, as the boundaries of the diagnosis have been debated (e.g., Andrews et al., 2010). For example, the symptom measure GAD-7 is sometimes often used as a broad indicator of anxiety symptoms in general, not as a measure of GAD per se. However, while the core symptom of worry is not specific to GAD, there are multiple lines of argument in support of GAD as a stand-alone disorder (Newman et al., 2013).

Additionally, focused treatments could have other advantages. Treatments that include several components may have the advantage being able to target a broad range of symptoms and comorbid disorders (Schaeuffele et al., 2020). On the other hand, the approach could also pose a challenge for both patients and clinicians to sufficiently target the core worry symptoms in GAD, which may have consequences for acceptability. Notably in routine care ICBT-studies, Ritola et al. (2022) and Mewton et al. (2012) observed a relatively high rate of drop-out: 44 % and 55 % classified as full completers. It has been argued that multi-component protocols are common because the incentive when developing is to add any plausibly effective components to increase the odds of a significant effect, often at the expense of considerations of how resource intensive the protocol is (Collins et al., 2024). This is relevant since qualitative studies of adverse effects of ICBT have found that many patients find the format stressful because of time demands (Gericke et al., 2021; Rozental et al., 2015). Thus, there is room for further improvement and treatment innovation, one of which could be that treatments based around a single theoretical model can potentially be more focused and condensed in terms of (for example) length, number of homework assignments and range of intervention strategies, which in turn could potentially increase acceptability and treatment credibility. Finally, condensed and focused treatments may potentially suit some patients better. For example, Newman and Fisher (2013) found that individuals with longer duration of GAD symptoms responded better to focused treatment (Cognitive Therapy or Systematic Desensitization) than a mixed treatment (both components at the same time).

In the case of GAD, a focused treatment would specifically target the worry process and its maintaining factors. One influential framework of worry in GAD is the Intolerance of Uncertainty Model (IUM) (Dugas et al., 1998). The key concept in this model is that individuals with GAD experience situations associated with uncertainty as highly aversive, and that this intolerance maintains the worrying over time. To reduce the anxiety associated with this uncertainty they engage in worrying, excessive control and avoidance behaviors. The IUM also incorporates other constructs such as positive beliefs about worry, negative problem orientation, and cognitive avoidance, but the core target in IU-treatment is to increase the individual's tolerance of uncertainty (Hebert and Dugas, 2019). IU-focused therapy has previously been shown to be effective when provided in traditional face-to-face format (Dugas et al., 2010; Dugas et al., 2022; Ladouceur et al., 2000). Although a number of ICBT protocols have included aspects of the IUM, we are not aware of any studies evaluating the acceptability and effectiveness of a construct-focused online IU-treatment for adults with GAD.

### 1.1 Aim

The aim with this study was to test a relatively short and construct-focused treatment for patients with GAD and evaluate the acceptability and feasibility when provided in within regular care at the Internet Psychiatry Clinic in Stockholm, as well as investigating preliminary effectiveness. If successful the protocol was to be used in future research into treatment mechanisms. Our hypothesis was that it would be feasible to adopt this protocol in this clinical setting and that the within-group effect would show a moderate to large reduction in self-rated GAD symptoms.

## 2 Material and method

### 2.1 Study setting

The study was conducted at the Internet Psychiatry Clinic in Stockholm (henceforth “the clinic”). The clinic has delivered evidence based ICBT treatments in routine psychiatric care since 2007 and accepts patients from all over Sweden. Patients usually self-refer online but they can also be referred from other health care providers. The clinic already offers treatments for depression, health anxiety, social anxiety disorder, panic disorder, insomnia and IBS. The trial was approved by the Swedish Ethical Review Authority (registration number: 2022-02380-01).

The online treatments provided at the clinic are based around text-modules with accompanying home-work exercises and asynchronous messages with patients on a designated webpage. When a patient has completed the exercises and answered questions about the module content, the psychologist responds with feedback, encouragement and answers any questions. The patients are encouraged to message the psychologist at any time in case of questions. For patient safety reasons, inactive patients (defined as no treatment activity for seven days) and patients with high ratings on depression and suicidal ideation are contacted by their psychologist.

Within this study, patients were treated by licenced psychologists. All psychologists had participated in a three-hour training seminar about the treatment protocol and the clinical assessment of GAD held by the authors, and had 30 min of weekly supervision during the trial to ensure fidelity in treatment and the assessment of GAD.

### 2.2 Participants and procedure

The inclusion/exclusion criteria and assessment procedure described mimic the normal routine for the clinic.

#### 2.2.1 Inclusion criteria

Patients were required to be (a) at least 18 years old; (b) read and speak Swedish well enough to be able to follow the text of the treatment protocol; (c) be able to use the internet platform, which requires electronic-ID for digital identification; (d) have the available time for participating in the 10-week treatment; (e) scoring at least 10 points on the GAD-7 scale (Spitzer et al., 2006) when filling out the initial registration form; (f) fulfil diagnostic criteria for GAD, judged by an assessing psychologist.

#### 2.2.2 Exclusion criteria

Comorbid psychiatric diagnoses were excluded only if determined to be contraindicated (for example psychosis, severe depression, or high risk of suicide), or if another treatment was judged to be prioritized (for example a patient with GAD but more severe symptoms of OCD). Patients with alcohol or substance abuse disorders were also excluded. When severe somatic health issues or psychosocial issues were judged likely to interfere with treatment, patients were also excluded.

#### 2.2.3 Recruitment and assessment

Individuals interested in participation in the study could get information about it and self-refer via the clinic's home page by filling out a registration form which included several screening questionnaires (listed in Section 2.4). Psychologists at the clinic could also recommend patients that did not initially seek treatment primarily for GAD to participate in the study, if they judged GAD to be a prominent and clinically significant feature of the patient's problem.

Eligible individuals received a video-call appointment with one of the psychologists at the clinic where the psychologist assessed GAD as well as common differential diagnoses (health anxiety, social anxiety disorder, depression and OCD) in addition to any other problem areas the patient had marked in their registration form. If another treatment at the clinic was deemed more likely to help with the patient's primary diagnosis, the psychologists could offer that treatment protocol instead. The psychologists used the patient's self-referral form as a basis for which additional parts of the M.I.N.I. (Sheehan et al., 1998) to administer, although ultimately their clinical judgement whether the patient fulfil DSM-5 criteria decided. If the assessment was unclear, the initial video call was supplemented with a telephone call after the psychologists had received supervision from a psychiatrist, or the researchers.

### 2.3 Intervention protocol

Our protocol was influenced by a treatment protocol for children and adolescents developed by Wahlund et al. (2020), based on the Intolerance of Uncertainty Model (Dugas et al., 1998). The treatment protocol was readapted for adults and to match the routines at the Internet Psychiatry Clinic. The treatment consisted of 8 text-based modules to be completed during a 10-week period. It mainly focused on how intolerance of uncertainty is the cause of excessive worry, and changing beliefs about uncertainty through changing worry-related safety-behaviors in behavioral experiments. See Table 1 for a brief description of the modules and associated exercises. Throughout treatment all exercises and rationales centre around the idea that intolerance of uncertainty is the main cause of excessive worry. While in no way a brief intervention, the protocol was shorter relative to other treatments at the clinic in terms of nr of modules (8 instead of 10) and amount of text. Mean wordcount for other protocols offered at the clinic is roughly 45,200, while this protocol has ca 26700.

**Table 1:** Content of protocol modules.

Module number	Module focus	Exercises/homework	Behavioral experiment
1	Learn about worry, intolerance of uncertainty, and the treatment platform	Worry diary	
2	Learn about worry behaviors and solvable/unsolvable worry thoughts	Expanded worry diary	
3	Learn about changing worry behaviors with explorative behavioral experiments	Expanded worry diary Initial behavioral experiment	Yes
4	Learn about changing intolerance of uncertainty through focused behavioral experiments	Focused behavioral experiments	Yes
5	Learn about active problem solving for solvable worry thoughts	Active problem solving Focused behavioral experiment	Yes
6	Learn about imaginal exposure for the uncertainty in unsolvable worry	Imaginal exposure Focused behavioral experiment	Yes
7	Learn about positive beliefs about worry	Focused behavioral experiment (modified)	Yes
8	Relapse prevention	Relapse prevention worksheets	

### 2.4 Measures

#### 2.4.1 Acceptability and feasibility

When patients started module 3, an adjusted version of the treatment credibility scale (Devilly and Borkovec, 2000) was administered. The modified scale has previously been used in multiple published studies, for example recently in Österman et al. (2024), from the clinic and contains five questions rated on a scale from zero to ten so that the resulting sum ranges from 0 to 50. The questions are reworded to describe worry symptoms rather than generic symptomatology and in contrast to Devilly and Borkovec (2000) ask patients about what they believe or expect rather than what they feel and expect. We further assessed acceptability by drop-out rate, defined in accordance with clinic routines which are as follows: Patients who are inactive for more than one week (activity is defined as sending an in-platform message of completing the homework assignments for a module) are contacted by therapists first via in-platform messages, then SMS and finally phone calls. If a patient stays inactive for 21 days despite these efforts, treatment is terminated. Alternatively, treatments can be terminated if the patient asks for treatment to be terminated or the clinician makes the assessment that treatment needs to be terminated (for instance if referral to inpatient care is needed). Any treatment that was terminated ahead of schedule was defined as a drop-out. At the end of treatment patients filled out patient global impression of improvement (PGI—I), a question on overall satisfaction with treatment and a modified version of the Client Satisfaction Questionnaire (Larsen et al., 1979). Scores range from 8 to 32. Smith et al. (2014) suggest that scores of 8–13 should indicate poor satisfaction, 14–19 fair satisfaction, 20–25 good satisfaction, and 26–32 excellent satisfaction.

#### 2.4.2 Primary treatment outcome measure

**The Generalized Anxiety Disorder 7-item scale (GAD-7)** is a well-established measure of generalized anxiety symptoms (Spitzer et al., 2006). Patients rate the frequency of a series of symptoms during the last two weeks, from 0 to 3 (0 = “not at all”, 1 = “several days”, 2 = “more than half the days”, 3 = “nearly every day”). A higher score represents more GAD-symptoms. The scale has been found to have good internal consistency (Cronbach α = 0.92) and test-retest reliability (intraclass correlation = 0.83) (Spitzer et al., 2006). A clinical cut-off of 10 or greater has been shown to have good sensitivity and specificity (Spitzer et al., 2006).

#### 2.4.3 Additional measures of comorbidity

Several measures are included in the clinics registration form and were used to assess comorbid symptoms and burden of illness at admission. These include the Panic Disorder Severity Scale – Self Rated (Houck et al., 2002), the Social Phobia Inventory (Connor et al., 2000), Liebowitz Social Anxiety Scale – Self Report (Heimberg et al., 1999), Short Health Anxiety Inventory-14 (Salkovskis et al., 2002), Alcohol Use Disorders Identification Test (Bergman and Källmén, 2002), Drug Use Disorders Identification Test (Berman et al., 2005), Insomnia Severity Index – 5 item version (Bastien et al., 2001), Adult ADHD Self-Report Scale (Kessler et al., 2005), World Health Organization Disability Assessment Schedule 2.0–12 question version (Ustun et al., 2010), Montgomery Åsberg Depression Rating Scale – Self Rated (Cunningham et al., 2011; Svanborg and Åsberg, 1994), Patient Health Questionnaire (Kroenke et al., 2001).

#### 2.4.4 Secondary treatment outcomes

The Penn-State Worry Questionnaire (PSWQ) is a well-established measure of excessive worry (Meyer et al., 1990). It has been shown to have good internal consistency in clinical samples (Cronbach α = 0.86–0.96) (Brown et al., 1992). Patients filled out the questionnaire at the beginning and end of the treatment period.

Patients also filled out weekly ratings on the short form of the intolerance uncertainty scale (IUS-12) (Carleton et al., 2007) to monitor change in IU and the Montgomery Åsberg Depression Rating Scale (MADRS-S) to monitor depression and suicidal ideation.

### 2.5 Data collection and statistical analysis

All questionnaires were administered online on the same platform patients engaged with the treatment protocol. Demographic data was collected from the patients' initial registration form. When logging in for the first time, patients had to fill out start-of-treatment questionnaires before continuing. For the rest of the treatment, patients filled in weekly questionnaires (GAD-7, IUS-12, and MADRS-S) the first time they logged in each week. Additional post-treatment questionnaires were administered during the last week of treatment.

The treatment effect on GAD-7 was estimated with a repeated measures linear mixed model, with random intercepts and effects for patients and a fixed effect of time (weeks). Analyses were conducted in R (R Core Team, 2023) using the lme4 package (Bates et al., 2015). The effect size was converted into Cohen's *d* for interpretability (Feingold, 2013) (formula nr 9, using standard deviation at week one). Missing data was treated as missing at random (Rubin, 1975). Changes in secondary outcomes were analysed with similar mixed effects models for the weekly measures and with *t*-tests for pre-post data. Effect size estimates for pre-post tests used pooled standard deviation. All presented confidence intervals are 95 %.

## 3 Results

### 3.1 Baseline characteristics

**Fig. 1:**
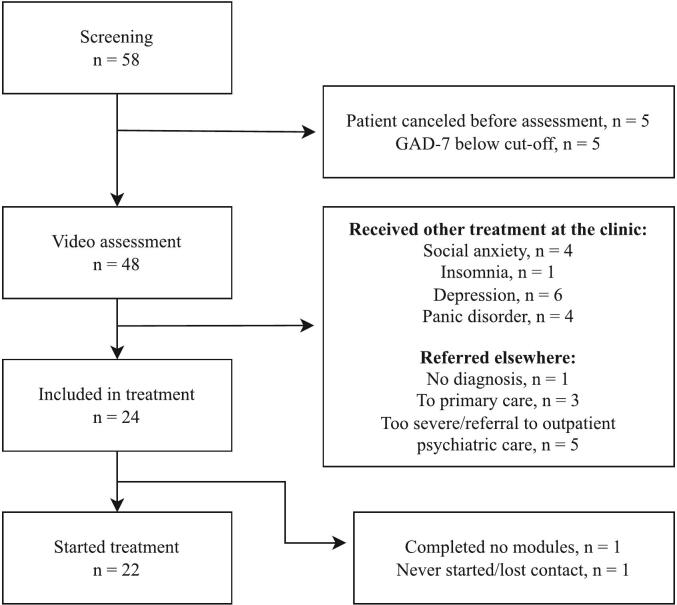
Patient flow-chart.

As seen in Fig. 1, fifty-eight individuals registered as interested in the participation. Of these ten were excluded before the initial video call (five registered interest but did not meet inclusion criteria and five withdrew before the assessment for unspecified reasons). The most common reason for exclusion was receiving another treatment at the clinic. Twenty-four patients were included in the study and 22 started the treatment. The majority of patients (19 out of 22) were women (legal gender based on Swedish personal identity number). Mean age was 33.8 (*SD* = 9.4). Table 2 presents additional baseline demographics.

**Table 2:** Baseline demographics.

Characteristic		N	Percent
Occupational status	Working	20	90.9
	Student	2	9.1
	Other	3	13.6
Source of income	Work	20	90.9
	Sickness benefit	4	18.2
	Support from family	1	4.5
	Other	3	13.6
How the patient found the study	Primary care	5	22.7
	National helpline (1177)	6	27.3
	Clinic homepage	2	9.1
	Other	9	40.9
Marital Status	Married/cohabiting partner	20	90.9
	Single	2	9.1
Education level	High-school	2	9.1
	Incomplete university	2	9.1
	Completed university (3 years)	2	9.1
	Completed university (> 3 years)	16	72.7
Children	Yes	8	36.4
	No	14	63.6
Previous or current medication for psychiatric condition	Yes	12	54.5
	No	10	45.5
Previous psychological treatment	Yes	18	81.8
	No	4	18.2
Previous CBT treatment	Yes	7	31.8
	No	15	68.2

Table 3 shows mean and standard deviations for self-rated questionnaires pre-treatment. The majority of the included patients showed a high degree of comorbid symptoms according to their self-ratings at screening. At the screening, 21 patients (95 %) scored above the cut-off for mild depression on MADRS-S, and 12 (55 %) scored above the cut-off for moderate depression. There was a high degree of self-rated symptoms of other anxiety disorders; panic disorder, social anxiety disorder and illness anxiety. Eight (36 %) had a positive screening on ASRS which screens for potential ADHD.

**Table 3:** Self-ratings at screening.

Scale	Mean	SD
PDSS	8.4	6.6
SPIN	22.1	13.3
LSAS	45.5	30.1
SHAI14	19.1	7.9
ISI	12.0	6.4
GAD7	16.2	3.1
PHQ9	12.9	6.0
MADRS-S	21.4	7.4
AUDIT	2.9	2.2
DUDIT	0.0	0.0
ASRS	2.7	2.0
WHODAS sum score	13.0	8.6

*Note.* PDSS = Panic Disorder Severity Scale, SPIN = the Social Phobia Inventory, LSAS = Liebowitz Social Anxiety Scale, SHAI = Short Health Anxiety Inventory-14, ISI = Insomnia Severity Index, GAD7 = Generalized Anxiety Disorder Scale-7, PHQ9 = Patient Health Questionnaire, MADRS-S = Montgomery-Åsberg Depression Rating Scale – Self Rated, AUDIT = Alcohol Use Disorders Identification Test, DUDIT = Drug Use Disorders Identification Test, ASRS = Adult ADHD Self-Report, WHODAS = World Health Organization Disability Assessment Schedule 2.0.

### 3.2 Primary outcomes: acceptability and effectiveness

The vast majority (90 %) of patients reported a global impression of improvement (feeling slightly to very much better compared to treatment start), and 85 % of patients rated themselves as satisfied or very satisfied with the treatment. Mean scores on the Client Satisfaction Questionnaire were high, *M* = 26.3, *SD* = 4.3. Furthermore, patients reported high mean scores on the Treatment Credibility Scale, administered three modules into the treatment, *M* = 36.9, *SD* = 6.4. We had a low rate of data attrition and drop-out, two patients dropped out and 20 out of 22 patients filled out post-treatment measurements. No adverse events were reported by clinicians. However, when analysing the data, we noted that one completer patient's symptoms got worse during treatment.

Patients completed a median of 6.5 modules (7.5 excluding dropout). Psychologists messaged patients on average 8 times and commented on average 7 times. Patients messaged psychologists on average 5 times during the treatment. This comparably lower rate is to be expected as much of the communication from the patients does not happen through messages but through filled out work-sheets.

**Fig. 2:**
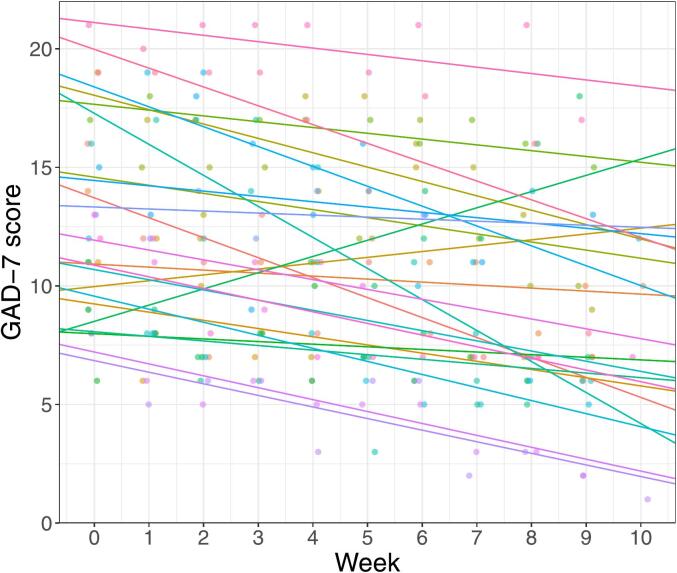
Self-rated GAD-7 symptoms by weeks in treatment. *Note.* Differing colours of dots and lines represent ratings and random effects for different patients. Jitter added to plot to avoid overlapping points.

Furthermore, we found a statistically significant decrease in self-rated GAD-symptoms based on the weekly ratings, *β* = −0.38, CI[−0.58, −0.13], *t*(20.49) = −3.38, *p* = 0.003. The fixed effect intercept at week one was 12.7 and estimated score at end of treatment was 9. The change corresponds to a Cohen's *d* = −0.92, CI[0.39, 1.46]. Fig. 2 shows self-ratings and random intercepts for study patients. See Table 4. for mean scores at screen, start of treatment and post treatment.

**Table 4:** Raw score change in self-ratings.

	Screening	Pre	Post	Pre-post change	Screen-post change
GAD-7	16.2 (3.1)	13 (4.1)	8.7 (4.6)	4.3	7.5
MADRS-S	21.4 (7.4)	18.05 (7.8)	12.6 (7.0)	5.4	8.8
IUS-12	–	42.2 (8.7)	35.5 (10.4)	6.7	–
WHODAS 2.0	13.0 (8.6)	16.1 (7.8)	11.9 (8.1)	4.2	1.1

*Note.* Pre means self-rating during the first week of treatment and post means during the tenth week of treatment. SD in parentheses. Pre-post change and screen-post change is raw difference.

### 3.3 Secondary outcomes

A statistically significant decrease in comorbid depressive symptoms based on weekly measures were found, *β* = −0.59, CI[−0.88, −0.29], *t*(19.12) = −4.02, *p* < 0.001, corresponding to a Cohen's *d* = 0.76, CI[0.37, 1.13]. There was also a significant on the PHQ-9, mean change *M* = 2.38, CI[0.30, 3.46], Hedge's *g* = 0.39, CI[0.03, 0.76], *p* = 0.03.

We also found a statistically significant decrease in intolerance of uncertainty, *β* = −0.60, CI[−0.98, −0.22], *t*(19.04) = −3.20, *p* = 0.005, corresponding to a Cohen's *d* = 0.7, CI[0.31, 1.17], and in trait worry measured on the PSWQ *M* = 5.81, CI[1.88, 9.73], Hedge's *g* = 0.76, CI[0.18, 1.35], *p* = 0.006. Lastly, there was a statistically significant improvement in patient ratings of disability, *t*(20) = 3.46, *p* = 0.002, corresponding to a Hedge's *g* = 0.51, CI[0.17, 0.85].

## 4 Discussion

Our results suggest that this relatively slimmed down, intolerance of uncertainty focused treatment feasible to implement within routine clinical care, acceptable to patients and preliminarily effective in reducing symptoms of GAD. This construct focused treatment had a low rate of drop-out, and a good level of engagement with the treatment in terms of median number of modules completed. Although the authors are satisfied with this level of drop-out and engagement, we have no indication of superiority compared to other ICBT treatments for GAD in routine care.

We saw generally positive ratings of client satisfaction and treatment credibility. Recruitment of patients finished within the planned time frame, and the ratio of inclusions mirrors the other treatments within the clinic as well as research on ICBT in general, indicating that we were able to reach the target population. We interpret these results as promising and warrants further investigation in larger randomized controlled trials.

The standardized within-group effect size of our primary outcome measure is comparable to what has been found in a meta-analysis of the effectiveness of face-to-face CBT when implemented in clinical care: For example, the within-group effect in Stewart and Chambless (2009) meta-analysis end up at exactly the same number as this study Cohen's *d* = 0.92, although since this is a small pilot study the estimate should be regarded as preliminary.

### 4.1 Strengths and limitations

A strength of this study is that we tested the protocol under conditions of high ecological validity. The protocol was piloted within the clinic it may eventually be implemented in, and our screening and assessment procedure ensures that individuals included were clinically diagnosable GAD-patients. This second point bears further emphasis because differences in recruitment, inclusion, and assessment procedure could be especially relevant when it comes to GAD. This is because anxiety and worry are common features of many clinical conditions, but excessive worry in GAD specifically is marked by its *chronic course* – for example increasing the duration criterion in DSM-III-R and DSM-IV was done to better differentiate GAD from worry as a symptom of depression (Crocq, 2017). To ensure that we were treating GAD and not a mixed sample of some patients with GAD and some with more vague presentations of anxiety or other anxiety conditions, we chose a somewhat higher cut-off on the GAD-7 scale when screening self-referrals – 10, compared to 8 (Ritola et al., 2022) or 5 (Hadjistavropoulos et al., 2014). We also instructed assessing psychologists were to make an integrated assessment of each patient case using DSM-5 criteria for GAD, as well as consider whether GAD was the primary treatment target or whether another program at the clinic would fit better. We see the high self-ratings on comorbid disorders as indication that the treatment program was successfully targeting the intended patient population (see Table 3)

Even so, readers should be cautious to generalize to other contexts: While all patients had clinically diagnosed GAD, it is noteworthy that the majority of the sample had a relatively high education degree with high rates of employment. This pattern is common for studies of ICBT patients as well as within routine care ICBT (Titov et al., 2018), but may limit the generalizability to patients in other contexts.

One potential strength in our theory-driven and construct focused protocol is that it is advantageous when investigating proposed mechanisms of change (Holmes et al., 2018). We found significant reductions in the target construct (intolerance of uncertainty), which we consider promising for future research. However, our small sample size limits our ability to detect interaction effects. Future research could further investigate mechanisms by comparing effectiveness to other theory-driven protocols for GAD within an RCT, in a larger sample. Further, testing moderation and mediated moderation of the treatment effect for target constructs when comparing two treatments could be an informative way to verify whether the protocols achieve change in the way that the respective theory predicts. Ideally the comparison should be to a treatment of similar length and demandingness but based on a different theoretical model.

One possible misgiving about more focused protocols is that giving patients fewer tools, e.g. fewer different concepts to make sense of their disorder and less varied exercises, may risk disadvantaging some patients. However, we do at least find preliminary evidence that the concepts that were “left out” of this relatively focused treatment did not cause the treatment to be completely ineffective. If this preserved clinical effectiveness is further validated in future studies, the scientific value of theory-driven construct-focused interventions lies in how they can be ethically applied to patients while also opening up for better investigation into the underlying theories. To clarify, the protocol is not a single component or brief intervention, and we have no evidence that the focus on one specific theoretical model made the treatment less demanding for patients. While the treatment protocol is shorter than other protocols at the clinic, there is significant range among treatments offered at other ICBT clinics around the world (Titov et al., 2018). Reporting on this is unfortunately often unclear and different treatment formats can make this comparison challenging.

The study has several limitations. First of all, no waiting list control group or comparison to treatment as usual was possible since it was a new protocol piloted on patients within the national healthcare system. This of course precludes a causal interpretation of the treatment effect. Secondly, the small sample size means there is significant uncertainty around the estimated effect size. Thus, the exact estimate is best regarded as preliminary. Furthermore, the missingness at random assumption could be regarded as problematic, which may yield biased estimates, especially with regards to our pre-post *t*-tests for secondary outcomes which use complete case analysis. However, since our rate of data-attrition was satisfactory with 2 out of 22 missing post-treatment measurements, this is unlikely to result in too severe misestimation. Thirdly, a further limitation relates to the potential non-specificity of the primary outcome measure GAD-7, capturing non-specific anxiety rather than symptoms that are a consequence of generalized excessive worry. However, as all our patients are diagnosed with GAD in accordance with best practices, we consider it reasonable to interpret the patients' self-report as a measure of their GAD symptomatology. Finally, our means of ensuring treatment adherence among treating psychologists was limited to weekly supervision (via video group call). This relatively light form of supervision was chosen to not interfere too much with ordinary clinic routines, and thus maintain the ecological validity of our findings and provide a clearer picture of the potential for future implementation in routine care. Future studies could potentially use text-data to retrospectively investigate treatment adherence without significantly interfering in clinical routines.

### 4.2 Conclusion

In conclusion, we find that this relatively short and focused internet treatment, based on the intolerance of uncertainty model of worry, was feasible, acceptable and preliminarily effective in routine psychiatric care. Future research should replicate this effect in larger samples and investigate the proposed mechanisms of change in these types of treatments.

## Funding

This work was supported by Region Stockholm via the Center for Innovative Medicine – CIMED (FoUI-963433) and ALF-Medicin project funding (FoUI-964186, FoUI-973842, FoUI-987509), and by 10.13039/501100008586Stiftelsen Bror Gadelius Minnesfond.

## Declaration of competing interest

The authors declare that they have no known competing financial interests or personal relationships that could have appeared to influence the work reported in this paper.

## Data Availability

In order to comply with Swedish and EU laws regulating protection of identifiable individuals, data from this trial cannot be shared by the authors. R scripts used for data analyses can be found at https://osf.io/8cz5k/
